# Phenological evolution in annual plants under light competition, changes in the growth season and mass loss

**DOI:** 10.1002/ece3.11294

**Published:** 2024-04-16

**Authors:** Willian T. A. F. Silva, Mats Hansson, Jacob Johansson

**Affiliations:** ^1^ Centre for Environmental and Climate Science Lund University Lund Sweden; ^2^ Department of Aquatic Resources Swedish University of Agricultural Sciences Lysekil Sweden; ^3^ Department of Biology Lund University Lund Sweden

**Keywords:** agriculture, flowering time, global warming, phenology, plant growth

## Abstract

Flowering time is an important phenological trait in plants and a critical determinant of the success of pollination and fruit or seed development, with immense significance for agriculture as it directly affects crop yield and overall food production. Shifts in the growth season, changes in the growth season duration and changes in the production rate are environmental processes (potentially linked to climate change) that can lead to changes in flowering time in the long‐term due to selection. In contrast, biomass loss (due to, for example, herbivory or diseases) can have profound consequences for plant mass production and food security. We model the effects of these environmental processes on the flowering time evolutionarily stable strategy (ESS) of annual plants and the potential consequences for reproductive output. Our model recapitulates previous theoretical results linked to climate change and light competition and makes novel predictions about the effects of biomass loss on the evolution of flowering time. Our analysis elucidates how both the magnitude and direction of the evolutionary response can depend on whether biomass loss occurs during the earlier vegetative phase or during the later reproductive phase and on whether or not plants are adapted to grow in dense, competitive environments. Specifically, light competition generates an asymetric effect of mass loss on flowering time even when loss is indiscriminate (equal rates), with vegetative mass loss having a stronger effect on flowering time (resulting in greater ESS change) and final reproductive output.

## Graphical abstract

Flowering time is an important phenological trait in plants. We explore the effects of growth season changes and biomass loss on the evolution of flowering time in annual plants and the potential consequences for biomass production.

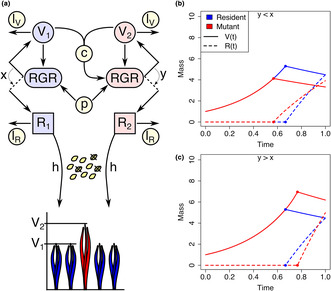

## 1 INTRODUCTION

Phenological traits are fundamental in determining the growth and ecological success of plant populations (Iler et al., 2021; Keller & Shea, 2021). In annual plants, in particular, the timing of allocation of resources from vegetative mass production to reproduction plays an important role in determining the reproductive output of an individual plant. Because of the photosynthetic process, the acquisition of energy in plants largely depends on their vegetative mass (photosynthetic tissues), which imposes a trade‐off between the production of such tissues and the production of tissues specialized in reproduction (Roff, 2000; Tuller et al., 2018; Williams, 1966). Therefore, the evolution of phenological traits, such as flowering time, is largely driven by environmental factors that affect the photosynthetic rate (e.g., light intensity, temperature, season duration). With that, we model the effects of light competition, changes in the growth season and mass loss on the evolution of flowering time in annual plants. We also suggest potential real‐world processes that can be represented by our model (e.g., climate‐driven rise in temperature can be represented by an increase in the basal production rate, herbivore activity and/or disease‐driven tissue necrosis can be represented by mass loss).

Changes in the growth season and environmental conditions can be caused by the ongoing global climate change (Christiansen et al., 2011; Kukal & Irmak, 2018) and affect the evolution of phenological traits (Franks et al., 2007, 2014; Parmesan & Yohe, 2003). In order to maximize fitness and/or avoid extinction, populations need to adapt to those changes or have a highly plastic phenology (Anderson et al., 2012). Adjustments of phenological traits (in particular, flowering time) have been observed in many species of plants and associated with the effects of climate change (CaraDonna et al., 2014; Cleland et al., 2006, 2007; Franks et al., 2007; Parmesan & Yohe, 2003). Furthermore, populations that fail to adjust their flowering time to match current environmental conditions caused by climate change have been reported to decline in size (Willis et al., 2008), a process that can lead to extinction in the long term. Therefore, understanding the consequences of changes in the growth season (e.g., as a result of climate change or other environmental processes) is fundamental for predicting the evolutionary fate of natural populations and the potential effects on agricultural species.

In addition to the widely known abiotic effects of climate change such as the rise in atmospheric CO_2_ concentration, rise in temperature and the consequent extension of the growth season, there are indirect biotic effects that can lead to changes in plant growth and the evolution of plant phenology. More specifically, the spread of viral, bacterial and fungal pathogens driven by climate change (Burdon & Zhan, 2020; Chaloner et al., 2021; Velásquez et al., 2018) can lead to a reduction in plant productivity because of the effective loss of plant mass (e.g., non‐functional leaves, inviable seeds, tissue necrosis). The reduction in plant productivity due to pathogens has been observed, in particular, in crop plants (Berger et al., 2007; Gonçalves et al., 2005; Nogués et al., 2002) and poses a threat to global agriculture (Strange & Scott, 2005). Hence, the ecological and evolutionary consequences of photosynthetic mass loss caused by abiotic or biotic effects, including those caused by climate change, need to be investigated.

Dynamic energy allocation models are excellent tools to investigate life‐history processes that directly depend on energetic input from an environmental source, such as mass production in plants. Together with optimality theory (Parker & Smith, 1990; Smith, 1978), these models have been used to predict the direction of evolutionary change as well as evolutionarily stable strategies (ESS) in plant phenology, such as the optimal timing of reproduction (Cohen, 1971, 1976; Iwasa, 2000). Similarly, we previously used a game‐theoretical approach of plant growth and reproduction in a competitive scenario where individual plants compete for light under climate change conditions to determine the effect of competition and climate change on the flowering time ESS of annual plants (Silva et al., 2021). These models also allow for the exploration of effects at the ecological scale, which contribute to our understanding of how climate change can impact agriculture in the near future.

In this study, we expand our model to explore additional environmental processes (i.e., mass loss) affecting the flowering time ESS of annual plants and the potential consequences for biomass production. We model plant growth as a simple process containing two parts: production/loss of vegetative mass and production/loss of reproductive mass. In our model, vegetative mass represents the biomass of photosynthetic tissues that capture energy from the environment and convert it into vegetative or reproductive mass. Reproductive mass represents the biomass of non‐photosynthetic tissues that are used to produce seeds. Modeling those processes is an important step towards understanding the evolutionary and ecological consequences of environmental conditions (including climate change) to plant populations. More specifically, we aim to address the question of how mass loss can affect the flowering time ESS in a competitive scenario under environmental conditions that can represent climate change, complementing previous theoretical investigations.

## 2 MATERIALS AND METHODS

We extended the models of competitive growth in annual plants by Cohen (1976) (growth without competition) and Silva et al. (2021) (growth with competition) to include vegetative and reproductive mass loss during plant growth. In addition to the dynamics described in the previous models, we assume that plants can be exposed to an agent (e.g., predation, diseases, weather) that causes loss of vegetative and/or reproductive mass throughout growth. Our model considers individual plant growth in a competitive scenario where the relative growth rate of an individual plant depends on its vegetative mass and the vegetative mass of the individuals surrounding it (mean vegetative mass of the population) (Mäkelä, 1985). We assume an annual life cycle, with discrete generations, and derive the conditions for population extinction, mutant invasion and trait evolution.

### 2.1 Plant growth and energy allocation

In our model, the annual plant life cycle consists of a vegetative growth process and a reproductive growth process. While vegetative growth is the process of production of photosynthetic biomass, reproductive growth is the process of production of non‐photosynthetic biomass that will be converted into seeds. Throughout plant growth, energy acquired via photosynthesis is allocated to those two processes, such that a proportion *u* of the energy is allocated to vegetative growth and the remaining 1–*u* is allocated to reproductive growth. Following optimal control theory (Cohen, 1971, 1976) and as an approximation to the near optimal reproductive output observed in empirical data (King & Roughgarden, 1983), we assume that the allocation of resources occurs via bang‐bang control, that is, resources are either completely allocated to vegetative growth (*u* = 1) or completely allocated to reproductive growth (*u* = 0) at any time point during plant growth.

For the sake of consistency with the terminology used in previous studies (Cohen, 1971, 1976; Silva et al., 2021), we define flowering time as the moment of switch of allocation of resources from vegetative to reproductive growth, that is, the time of switch from *u* = 1 (vegetative growth) to *u* = 0 (reproductive growth). We study how flowering time can evolve through the invasion of a resident monomorphic population that flowers at time *x* by an initially rare mutant that flowers at time *y*, using an adaptive dynamics approach (Geritz et al., 1998). We model plant growth according to the following equations:

(1) $$\frac{dV_{i}}{\mathrm{dt}}=u_{i}\left(t\right)\cdot V_{i}\left(t\right)\cdot \left(p+c\cdot \left[V_{i}\left(t\right)-\bar{V\left(t\right)}\right]\right)-l_{V}\cdot V_{i}\left(t\right)$$

(2) $$\frac{dR_{i}}{\mathrm{dt}}=\left[1-u_{i}\left(t\right)\right]\cdot V_{i}\left(t\right)\cdot \left(p+c\cdot \left[V_{i}\left(t\right)-\bar{V\left(t\right)}\right]\right)-l_{R}\cdot R_{i}\left(t\right)$$

In Equations (1) and (2), the vegetative mass $V_{i}\left(t\right)$ and reproductive mass $R_{i}\left(t\right)$ of an individual plant *i* increase with a relative growth rate that depends on the basal production rate *p*, which represents the growth rate of individuals in a symmetric competition scenario (monomorphic population) and depends on climatic conditions, and an additional effect from asymetric competition when the size of an individual plant differs from the population mean size (see Appendix), with *c* representing the magnitude of the effect of asymetric competition. Additionally, vegetative and reproductive mass loss occur at constant rates $l_{V}$ and $l_{R}$, respectively.

**FIGURE 1:**
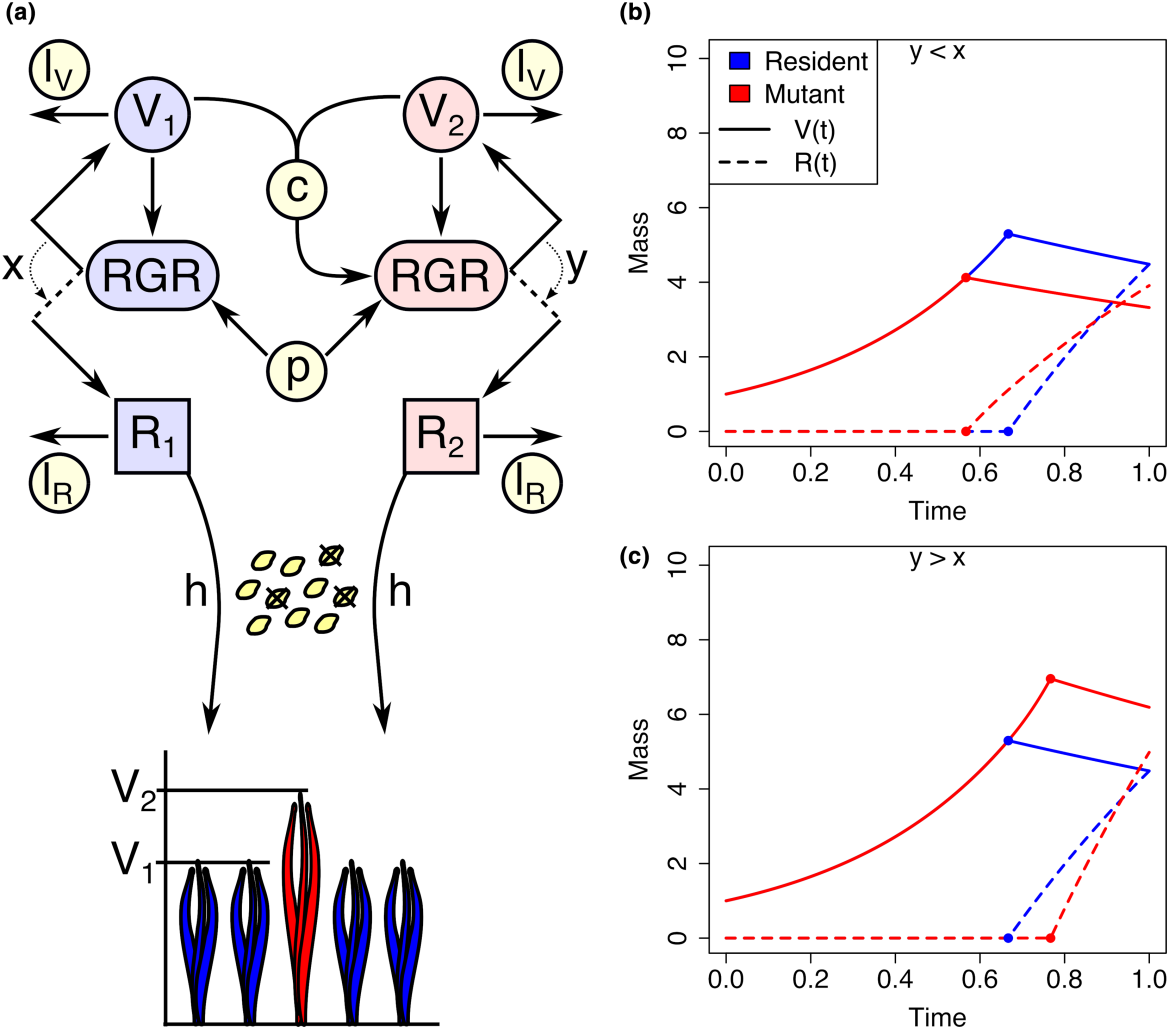
Model overview. (a) Individual growth consists of vegetative mass growth ($V_{i}$) and reproductive mass growth ($R_{i}$), which increase with vegetative biomass and the relative growth rate (RGR). The RGR is affected by climatic conditions (production rate *p*) and competition strength (*c*), and is allocated from vegetative mass growth to reproductive mass growth at time *t* = *x* in the resident population (subscript *i* = 1) and *t* = *y* in the invading mutant (subscript *i* = 2). The RGR of the resident strategy is unaffected by the mutant because of the mutant's low frequency. Vegetative and reproductive mass decrease at the rates *l*_*V*_ and *l*_*R*_, respectively, throughout plant growth. The final reproductive mass *R* (*E*) is converted into next‐generation seedlings by a conversion efficiency factor *h*. (b and c) Illustration of vegetative (solid curves) and reproductive (dashed curves) mass growth of an early‐flowering (b; *y* < *x*) and a late‐flowering (c; *y* > *x*) mutant (red) in a resident population (blue) at the monomorphic optimal flowering time, showing the switch from vegetative to reproductive mass production.

In our game theory approach, we assume that the resident population (subscript *i* = 1) is monomorphic with regard to flowering time (*x*) and large enough such that flowering time mutants (subscript *i* = 2) are rare and do not affect the mean flowering time of the population ($\bar{V\left(t\right)}=V_{1}\left(t\right)$; Figure 1a). It follows from Equations (1) and (2) that the growth dynamics of resident individual plants are represented by the following equations:

(3) $$\frac{dV_{1}}{\mathrm{dt}}=u_{1}\left(t\right)\cdot p\cdot V_{1}\left(t\right)-l_{V}\cdot V_{1}\left(t\right)$$

(4) $$\frac{dR_{1}}{\mathrm{dt}}=\left[1-u_{1}\left(t\right)\right]\cdot p\cdot V_{1}\left(t\right)-l_{R}\cdot R_{1}\left(t\right)$$

The relative growth rate of flowering time mutants, however, is affected by the resident flowering time and, therefore, the growth dynamics of a mutant individual plant are represented by the following equations:

(5) $$\frac{dV_{2}}{\mathrm{dt}}=u_{2}\left(t\right)\cdot V_{2}\left(t\right)\cdot \left(p+c\cdot \left[V_{2}\left(t\right)-V_{1}\left(t\right)\right]\right)-l_{V}\cdot V_{2}\left(t\right)$$

(6) $$\frac{dR_{2}}{\mathrm{dt}}=\left[1-u_{2}\left(t\right)\right]\cdot V_{2}\left(t\right)\cdot \left(p+c\cdot \left[V_{2}\left(t\right)-V_{1}\left(t\right)\right]\right)-l_{R}\cdot R_{2}\left(t\right)$$

The growth season is assumed to begin at a time point *B*, with plants assumed to be young seedlings that can produce biomass through photosynthesis ($V_{i}\left(B\right)=V_{B}$ and $R_{i}\left(B\right)=0$), and end at a time point *E*, representing the end of seed production. In order to represent the life cycle of an annual plant in the context of regular seasonal cycles, we assume that the length of the growth season (E−B) is equal to or shorter than the length of the year.

In a monomorphic population with regard to flowering time (Equations (3) and (4)), the total mass production rate is proportional to the vegetative mass and does not decrease over time, resulting in isometric rather than allometric scaling of the production rate. Additionally, we assume that there is no constraint to growth due to, for example, self‐shading or limited nutrient availability. The assumption of isometric scaling of the production rate results in the following alternative interpretations: (i) all cells composing the vegetative mass are photosynthetic and equally efficient at producing biomass; (ii) vegetative non‐photosynthetic cells boost the efficiency of vegetative photosynthetic cells (via, for example, the supply of nutrients) such that the lack of production by non‐photosynthetic cells is compensated for by an increased efficiency in photosynthesis and biomass production by photosynthetic cells; or (iii) vegetative photosynthetic and non‐photosynthetic cells occur in a fixed proportion.

In an asymmetric competition scenario, a rare mutant that flowers earlier than the resident population suffers a reduction in its relative growth rate due to shading by the still growing resident plants. Conversely, a rare mutant that flowers later than the resident population has an increased relative growth rate due to its still growing vegetative mass, while vegetative growth of resident plants is no longer happening. Because mutants are rare, they do not affect the mean relative growth rate of the resident population.

### 2.2 Invasion fitness

We use a simple population dynamics model with discrete generations in which the density (or population size) of adult plants is kept constant (*K*) via density‐dependent seedling survival when the reproductive output is above a certain threshold (see Appendix). We assume that mortality only occurs before the start of the growth season (when there is no biomass production via photosynthesis, a developmental stage referred to as skotomorphogenesis) and after the end of the growth season (when seeds have been dispersed and all adults die), that is, there is no mortality during plant growth through photosynthesis (photomorphogenesis). In other words, mortality is density‐dependent and does not depend on the growth strategy adopted by the individuals in the population.

The number of seedlings *W* produced by an individual plant, and thereby the growth rate of the resident and mutant populations, is proportional to its final reproductive mass $R_{i}\left(E\right)$ at the end of the growth season by a factor *h*, that is, $W=h\cdot R_{i}\left(E\right)$, which accounts for the effects of seed size and survival. The final reproductive mass $R_{i}\left(E\right)$ of an individual plant depends on its own flowering time as well as the flowering time *x* of the resident population, so we denote *W*(*y*,*x*) and *W*(*x*,*x*) the per capita number of seedlings produced by mutant (with flowering time *y*) and resident plants, respectively. Therefore, a positive growth rate of a small monomorphic population requires that $R_{i}\left(E\right)>1/h$, resulting in a yearly per capita production of seedlings greater than one. When $R_{i}\left(E\right)<1/h$, the population will decline and eventually go to extinction. Therefore, we set 1/*h* as the extinction threshold.

We further assume, for simplicity, that seeds do not survive across several growth seasons forming seed banks and, therefore, all seedlings in any given growth season (year) are produced by plants in the previous growth season, that is, seeds produced in a given generation do not compete with seeds produced in previous generations.

The invasion fitness of a rare mutant strategy with flowering time *y* in a resident population with flowering time *x* can be calculated as $s_{x}\left(y\right)=\frac{W\left(y,x\right)}{W\left(x,x\right)}$ (see Appendix). Given that the yearly per capita seedling production is proportional to $R_{i}\left(E\right)$ by a constant factor *h*, the invasion fitness of the mutant can be simplified and centered around zero by setting

(7) $$s_{x}\left(y\right)=\frac{R_{2}\left(E\right)}{R_{1}\left(E\right)}-1$$

Using the invasion fitness (Equation (7)), we can find the flowering time ESS by calculating the flowering time x* that satisfies the following condition:

(8) $${\left.\frac{ds_{x}\left(y\right)}{\mathrm{dy}}\right|}_{y=x}=0$$

Table 1 contains a brief description of all the parameters and variables used in the model analyses and simulations. All calculations were performed on R version 4.0.3 (R Core Team, 2017).

**TABLE 1:** Definitions of parameters and variables used in the model and simulations in alphabetical order.

Variable/parameter	Definition
“1”	Subscript indicating resident strategy parameters/variables
“2”	Subscript indicating mutant strategy parameters/variables
*B*	Beginning of the growth season (time point)
*c*	Strength of the effect caused by competition for light between mutant and resident strategies
*E*	End of the growth season (time point)
*h*	Conversion factor from reproductive mass to number of seedlings
*K*	Carrying capacity of the habitat
*l*_*V*_	Rate of vegetative mass loss
*l*_*R*_	Rate of reproductive mass loss
*p*	Basal mass production rate per unit vegetative mass
*R*	Reproductive mass
*R*_*B*_	Initial reproductive mass at the beginning of the growth season
*s*_*x*_ (*y*)	Invasion fitness (or selection coefficient) of the mutant strategy
*t*	Growth time
*u*	Fraction of net production allocated to vegetative growth
*V*	Vegetative mass (photosynthetic tissue)
*V*_*B*_	Initial vegetative mass at the beginning of the growth season
*W*	Number of seedlings
*W* (*x,x*)	Per capita number of seedlings produced by the resident strategy
*W* (*y,x*)	Per capita number of seedlings produced by the mutant strategy
*x*	Time of allocation of resources of the resident strategy
x*	Evolutionary stable strategy (ESS)
*y*	Time of allocation of resources of the mutant strategy

### 2.3 Environmental conditions

Environmental conditions are represented by the basal production rate *p* (climate‐dependent photosynthetic efficiency), the start (*B*) and end (*E*) of the growth season, the length of the growth season (*E*‐*B*) and the rates of vegetative mass loss $l_{V}$ and reproductive mass loss $l_{R}$. As in our previous study (Silva et al., 2021), we analyzed the effect of (i) an increased production rate by varying *p*, (ii) an advancement of the growth season by decreasing the values of *B* and *E* in parallel within the year and keeping the growth season length (*B*‐*E*) constant, (iii) an extension of the growth season length (*B*‐*E*) by simultaneously decreasing *B* and increasing *E* by the same amount of time while holding the season center (*B* + *E*)/2 constant. Additionally, we analyzed the effect of (iv) vegetative mass loss by increasing the value of $l_{V}$, (v) reproductive mass loss by increasing the value of $l_{R}$, and (vi) indiscriminate mass loss by increasing the values of $l_{V}$ and $l_{R}$ simultaneously by the same amount.

We analyzed the effects of the environmental conditions (i–vi) in the context of light competition by contrasting situations where competition is either present or absent, representing scenarios where plants grow densely together or more sparsely, respectively. We set *p* = 3, $l_{R}=0$, $l_{V}=0$, *B* = 0 and *E* = 1 as default parameter values representing historical climate and growth conditions. The presence of light competition was represented by setting *c* = 0.25, in contrast to *c* = 0 representing the absence of competition. The following parameters were assumed to be constant throughout the analyses: *V*_*B*_ = 1, *R*_*B*_ = 0, *h* = 1/*e* = 0.36. Stochastic variation in developmental and phenological traits (e.g., germination time) and climate conditions (e.g., production rate, season duration) is not explored in the current study.

## 3 RESULTS

### 3.1 Evolutionarily stable strategy

As previously shown (Silva et al., 2021), the growth pattern of the vegetative mass is the same in both resident and mutant strategies until either strategy flowers earlier that the other (Figure 1b,c), in which case, the early‐flowering strategy starts producing reproductive mass earlier but with a lower relative growth rate than the late‐flowering strategy during reproductive mass production. In any case, with a constant rate of vegetative mass loss ($l_{V}>0$), vegetative mass decreases steadily once individuals switch production to reproductive mass. During reproductive mass production, there is a gradual decrease in production due to the continued loss of vegetative mass and gradual loss of reproductive mass ($l_{R}>0$; Figure 1b,c).

We start by establishing that our model reproduces the same predictions as previous theory when either competition or mass loss is absent. The analyses of the invasion fitness and derivation of selection gradient (see Appendix) showed that the flowering time ESS is the value of x* that solves the general equation

(9) $$e^{x^{*}\cdot \left(l_{R}-l_{V}\right)}=c\cdot V_{B}\cdot e^{p\cdot x^{*}}\cdot \int_{x^{*}}^{E}e^{t\cdot \left(l_{R}-2\cdot l_{V}\right)}\;\mathrm{dt}+p\cdot \int_{x^{*}}^{E}e^{t\cdot \left(l_{R}-l_{V}\right)}\;\mathrm{dt}$$

Equation (9) is the general expression for the flowering time ESS resulting from our model but we show that it can be reduced to previously reported simpler expressions in the absence of mass loss or light competition. Note that in Equation (9) the integral terms are left unsolved in order to account for the situations where there is no mass loss ($l_{V}=l_{R}=0$) or mass loss is indiscriminate ($l_{R}=l_{V}$), otherwise the solution would be undefined in those particular cases.

**FIGURE 2:**
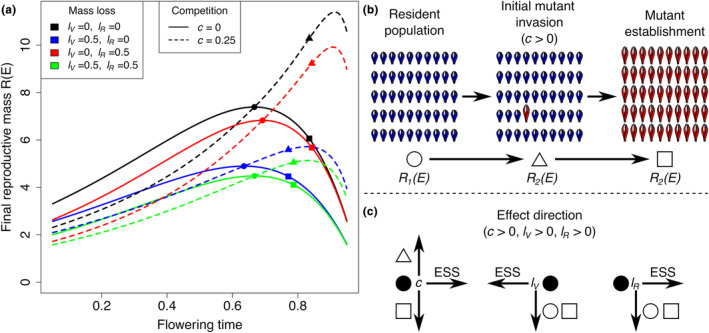
General effect of competition and mass loss on the ESS and its reproductive mass during initial invasion and after establishment as a resident population. (a) Fitness landscape of ESS strategies in monomorphic populations (solid lines) and populations under mutant invasion (dashed lines). Circles indicate the ESS's and their respective final reproductive mass in the absence of competition, triangles indicate the final reproductive mass of the competitive mutant ESS during initial invasion, and squares indicate the final reproductive mass of the mutant ESS once it becomes the resident population. (b) Illustration of the population states at the points indicated in the fitness landscape shown in (a). (c) Direction of the effect of competition, vegetative mass loss and reproductive mass loss on the ESS, relative to the basic model without competition or mass loss, solid black line in (a).

In the absence of mass loss ($l_{V}=l_{R}=0$) and light competition (*c* = 0), the singular point or, more specifically, the strategy that maximizes the final reproductive mass is defined as

(10) $$x^{*}=E-\frac{1}{p}$$

which corresponds to the previously reported (Cohen, 1971) optimal flowering time of a monomorphic population that is not affected by light competition or mass loss (Figure 2a).

In the presence of light competition (*c* > 0) and absence of mass loss ($l_{V}=l_{R}=0$), the singular point is the same found by Silva et al. (2021):

(11) $$x^{*}=E-\frac{1}{p+c\cdot V_{B}\cdot e^{p\cdot x^{*}}}$$

When competition does not play a role but mass loss (vegetative and/or reproductive) does, the singular point is defined as previously reported (Macevicz & Oster, 1976; Mirmirani & Oster, 1978):

(12) $$x^{*}=E-\frac{1}{l_{R}-l_{V}}\cdot \ln\left(\frac{p+l_{R}-l_{V}}{p}\right)$$

**FIGURE 3:**
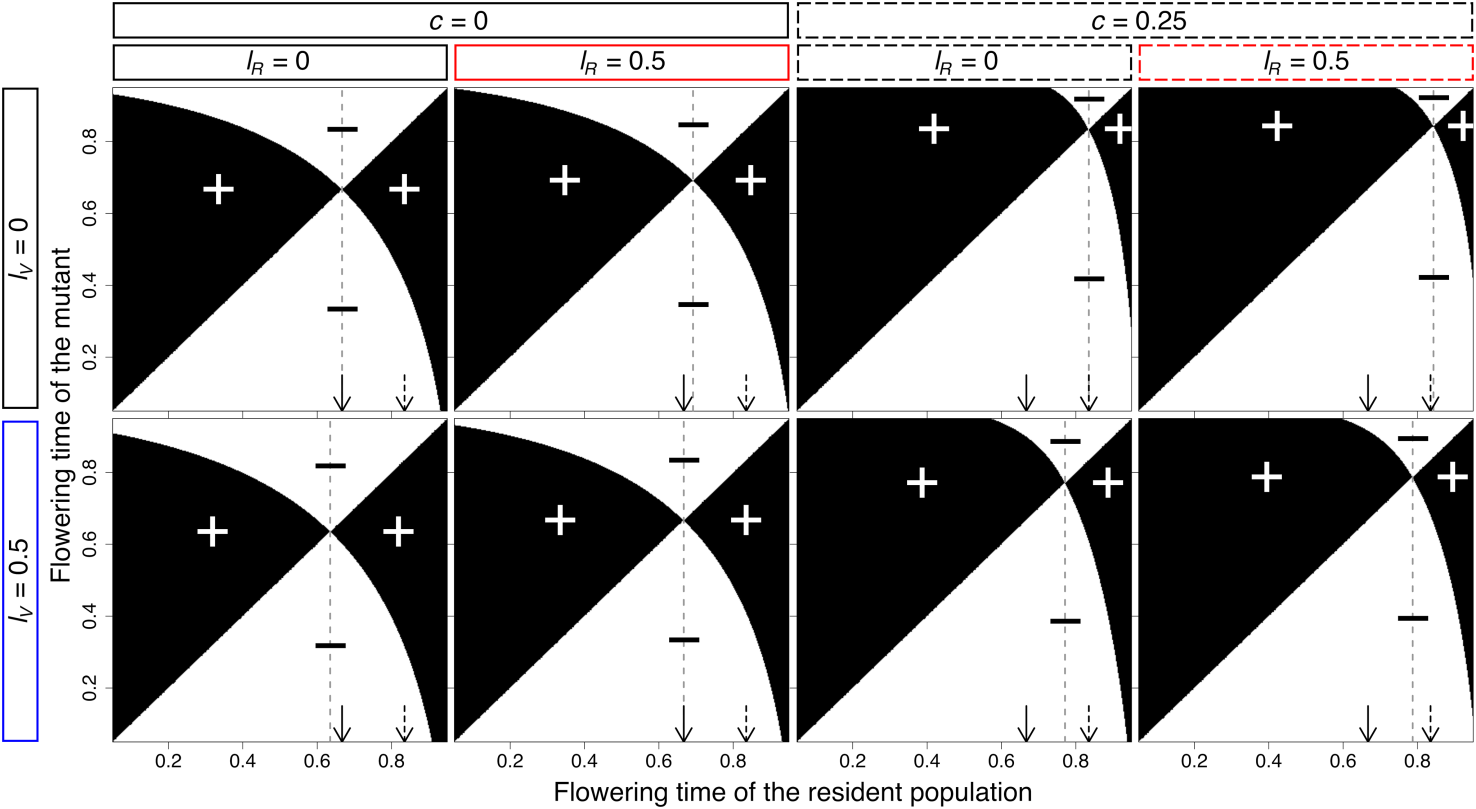
Paiwise invasibility plots (PIP) showing the ESS's (vertical dashed lines) and the invasion fitness, positive (+) or negative (−), of mutants under competitive (dashed rectangles) and mass loss conditions (blue and red rectangles). Solid and dashed arrows are references to the ESS's in the absence (first PIP) and presence (third PIP) of competition without mass loss, respectively.

**FIGURE 4:**
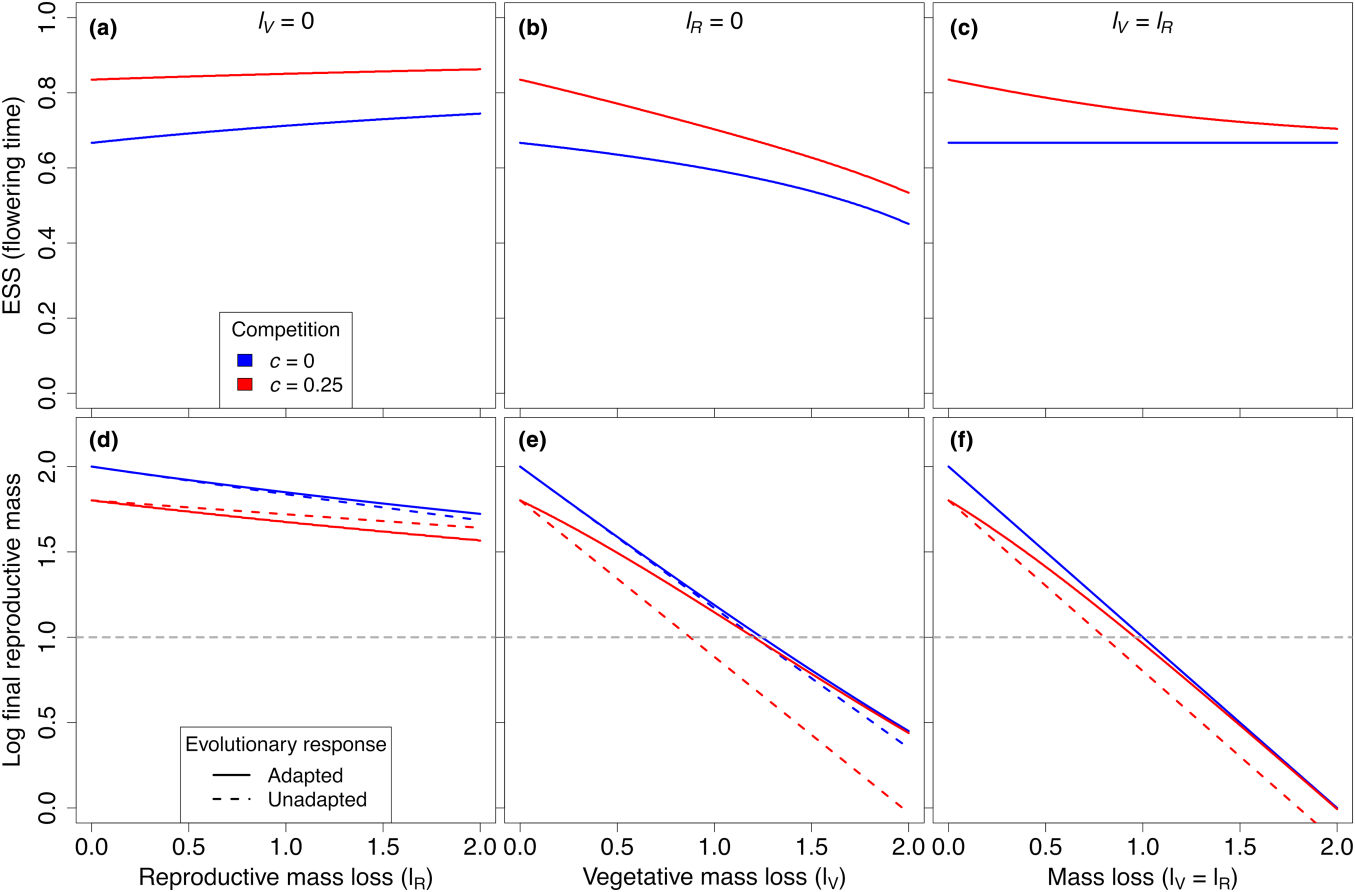
Changes in the ESS (a–c) and final reproductive mass *R*(*E*) (d–f) caused by reproductive (*l*_*R*_) and/or vegetative (*l*_*V*_) mass loss during plant growth, relative to a population that is not affected by mass loss ($l_{R}=l_{V}=0$). Solid curves in (d–f) indicate the final reproductive mass of the adapted population; dashed curves indicate the final reproductive mass when the population does not change its flowering time, i.e. remains adapted to the historical conditions (no mass loss). Gray dashed lines indicate the extinction threshold for *h* = 1/*e*.

We note from Equation (12) that when mass loss is restricted to either vegetative mass ($l_{R}=0$ and $l_{V}>0$) or reproductive mass ($l_{V}=0$ and $l_{R}>0$), we find that $\left|\frac{dx^{*}}{l_{V}}\right|>\left|\frac{dx^{*}}{l_{R}}\right|$ (a special consequence of the assumption of isometric scaling). This outcome is particularly unintuitive from a biological point of view because, without competition, indiscriminate mass loss ($l_{R}=l_{V}$) leads to vegetative mass loss canceling out the effect of reproductive mass loss ($l_{R}-l_{V}=0$; Equation (12)), and with that, there is no change in the ESS (Figures 3 and 4a–c, see $l_{V}=l_{R}$ with *c* = 0). However, when vegetative and reproductive mass loss are mutually exclusive, their effects differ not only in the direction of selection but also in the magnitude of selection because

(13) $$\left|\frac{d}{dl_{V}}\ln\left(\frac{p-l_{V}}{p}\right)\right|>\left|\frac{d}{dl_{R}}\ln\left(\frac{p+l_{R}}{p}\right)\right|$$

This difference in the magnitude of their isolated effects is more apparent when mass loss is high. When both vegetative and reproductive mass loss occur but at different rates ($l_{V}\neq l_{R}$ and $l_{V},l_{R}>0$), the direction of selection will depend on the difference between the rates of reproductive mass loss and vegetarive mass loss, with negative differences ($l_{R}-l_{V}<0$) leading to selection for earlier flowering and positive differences ($l_{R}-l_{V}>0$) leading to selection for later flowering.

Now we consider the occurrence of both light competition and mass loss simultaneously. From Equation (9) we note that, if $l_{R}\neq l_{V}$, the sigular point is the value of x* that solves the equation

(14) $$x^{*}=\frac{1}{l_{R}-l_{V}}\cdot \ln\left(c\cdot V_{B}\cdot e^{p\cdot x^{*}}\cdot \int_{x^{*}}^{E}e^{t\cdot \left(l_{R}-2\cdot l_{V}\right)}\;\mathrm{dt}+p\cdot \int_{x^{*}}^{E}e^{t\cdot \left(l_{R}-l_{V}\right)}\;\mathrm{dt}\right)$$

We note that the term containing the effect of competition introduces an asymmetry between vegetative and reproductive mass loss (i.e., $l_{R}-2\cdot l_{V}$). Because of that, in a competitive scenario (*c* > 0) with indiscriminate mass loss ($l_{R}=l_{V}$), vegetative mass loss has a stronger effect on the ESS than reproductive mass loss.

Given that $x^{*}>0$ always, we can infer from Equation (14) that

(15) $$\mathrm{sgn}\left[l_{R}-l_{V}\right]=\mathrm{sgn}\left[\ln\left(c\cdot V_{B}\cdot e^{p\cdot x^{*}}\cdot \int_{x^{*}}^{E}e^{t\cdot \left(l_{R}-2\cdot l_{V}\right)}\;\mathrm{dt}+p\cdot \int_{x^{*}}^{E}e^{t\cdot \left(l_{R}-l_{V}\right)}\;\mathrm{dt}\right)\right]$$

and, with that, we note that increasing the competition effect will always result in an increased absolute value of the logarithmic term in Equation (14). Therefore, we can conclude that, as in the simpler model without mass loss, we also obtain $\frac{dx^{*}}{\mathrm{dc}}>0$, that is, competition for light delays the flowering time ESS (Figure 2a–c). Additionally, an increased production rate also delays flowering time ($\frac{dx^{*}}{\mathrm{dp}}>0$).

Using the same approach and Equation (15), we again find that loss of vegetative mass results in an earlier flowering time ESS ($\frac{dx^{*}}{l_{V}}<0$) and loss of reproductive mass results in a later flowering time ESS ($\frac{dx^{*}}{l_{R}}>0$) (Figure 2a–c). Alternatively, setting $l=l_{R}-l_{V}$ and c=0, we obtain $\frac{dx^{*}}{l}>0$. As a result, loss of mass selects for flowering time in opposite directions depending on the relative magnitude of vegetative and reproductive mass loss, in addition to the asymmetry (i.e., $l_{R}-2\cdot l_{V}$) introduced by competition.

Pairwise invasibility plots show that the singular point x* is both evolutionarily and convergence stable (ESS; Figure 3). Additionally, they show that in the absence of competition, indiscriminate mass loss does not lead to a change in the ESS (Figure 3; compare $l_{V}=l_{R}$ when *c* = 0). When competition is present, indiscriminate mass loss leads to an earlier flowering time ESS because of the asymmetric effect introduced by competition that makes the effect of vegetative mass loss outweigh the effect of reproductive mass loss regarding the direction of change of the ESS (Figure 3; compare $l_{V}=l_{R}$ when *c* = 0.25).

### 3.2 Adaptation to mass loss

The effect of mass loss on the flowering time ESS depends on the type of mass loss (tissue‐specific or indiscriminate) and presence of competition (Figure 4a–c), as stated above. While mass loss always results in a smaller final reproductive mass, adaptation does not necessarily lead to an increased final reproductive mass (Figure 4d–f). In fact, when mass loss is restricted to reproductive mass in a competitive scenario, a population that remains adapted to the historical condition (no mass loss) increases its final reproductive mass relative to a population that adapts to reproductive mass loss by delaying its flowering time (Figure 4a,d).

### 3.3 Adaptation to changes in the growth season

In addition to the results mentioned above, we also explored the model in the context of the climate change scenarios explored by Silva et al. (2021). In those scenarios, climate change can affect the production rate, shift the growth season and/or change the duration of the growth season. While the general pattern resulting from those scenarios do not change, the existence of mass loss can change the direction of selection relative to equivalent scenarios without mass loss.

**FIGURE 5:**
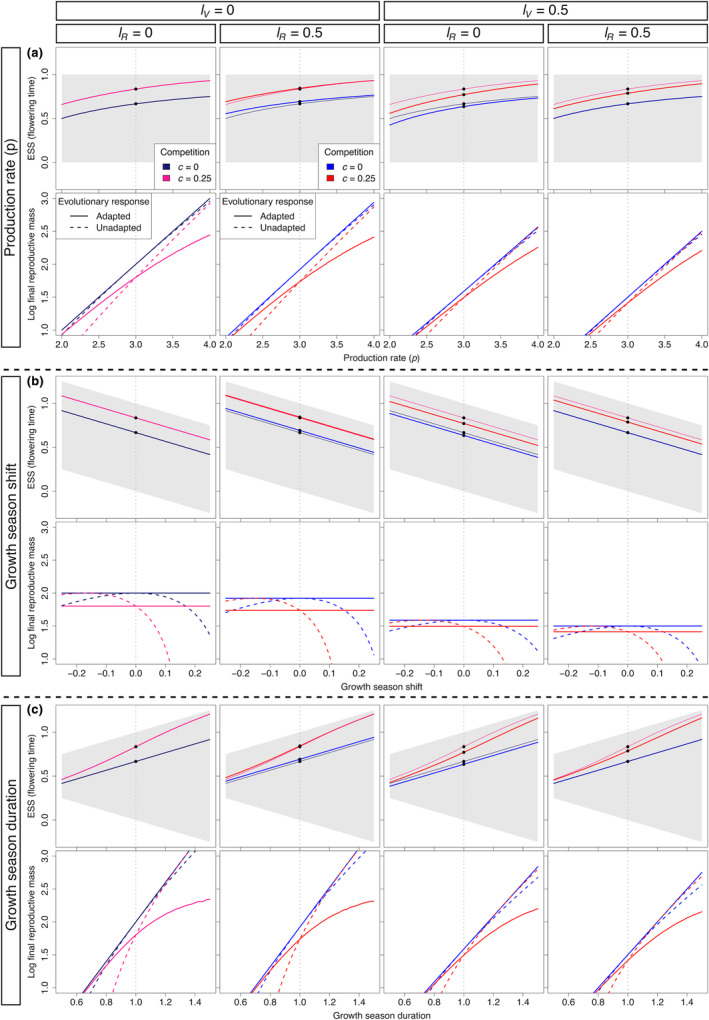
Changes in the ESS (top row of each panel) and final reproductive mass *R* (*E*) (bottom row of each panel) cause by (a) changes in the production rate, (b) shift in the growth season, or (c) changes in the duration of the growth season under different mass loss conditions (columns), relative to the default growth season (points; *p* = 3 and 0 < *t* < 1). Gray areas indicate the span of the growth season during the year (y‐axis). Vertical gray lines indicate default conditions (which can be interpreted as historical conditions). The final reproductive mass is shown for populations that adapt to climate change (solid curves) and populations that remain adapted to the historical conditions (unadapted; dashed curves). ESS curves for *l*_*V*_ = *l*_*R*_ = 0 (absence of mass loss; thin lines) are shown as references to different mass loss conditions.

Generally, an increase in the production rate selects for later flowering time. However, mass loss can select for earlier ($l_{V}>0$) or later ($l_{R}>0$) flowering time relative to a situation where loss is absent. As the production rate increases, reproductive mass loss tends to be compensated for faster than vegetative mass loss, in terms of changes in the ESS (Figure 5a). Similarly, an earlier start of the growth season selects for earlier flowering time (Figure 5b), while an extension of the growth season selects for later flowering time (Figure 5c). In all cases, light competition selects for later flowering time.

While changes in the growth season results in changes in the direction of selection of flowering time, they do not necessarily lead to changes in the final reproductive mass (e.g., seed production). In fact, when there is a shift in the growth season that is not followed by an increased production rate or extension of the season, the final reproductive mass remains constant even though flowering time changes due to selection. It is also important to note that, in the absence of light competition, adapting to environmental conditions via selection in flowering time always leads to higher reproductive mass production than retaining the historical flowering time. However, in the presence of competition and environmental conditions with an increase in production rate and extension of the growth season, an adapted population will produce a lower final reproductive mass than an unadapted population that keeps its historical flowering time (Figure 5a,c).

## 4 DISCUSSION

In our model, the evolution of flowering time, as represented by a change in the flowering time ESS relative to the historical flowering time in a monomorphic population, is driven by the effects of light competition, vegetative/reproductive mass loss and changes in the growth season (e.g., climate‐driven changes). We confirm results from previous theoretical work and propose a novel theoretical prediction regarding the joint effect of light competition and mass loss.

From previous theoretical studies, our model recapitulates that higher photosynthetic efficiency (represented by the basal production rate *p*) promotes selection for later flowering (Cohen, 1971). Similarly, light competition, in the absence of mass loss, also leads to selection for later flowering (Silva et al., 2021).

In the absence of competition, reproductive mass loss leads to selection for later flowering, while vegetative mass loss leads to selection for earlier flowering (Macevicz & Oster, 1976; Mirmirani & Oster, 1978). However, indiscriminate mass loss does not affect flowering time because rates of vegetative and reproductive mass loss balance each other.

In general, we found that environmental conditions in the form of increased biomass productivity (e.g., due to rising atmospheric CO_2_; Dusenge et al., 2019) or extended growth seasons result in selection for later flowering (Silva et al., 2021). However, it is important to note that, under light competition, flowering time adaptation to such environmental conditions does not necessarily lead to increased reproductive output (fitness maximization) relative to the historical output (Silva et al., 2021; Vincent et al., 1996).

In addition to the results mentioned earlier, our model also predicts novel evolutionary outcomes when light competition is assumed to be an inherent process in a dense plant population. Light competition generates an asymetric effect of mass loss on flowering time even when loss is indiscriminate (equal rates), with vegetative mass loss having a stronger effect on flowering time (resulting in greater ESS change) and final reproductive output. When mass loss is restricted to reproductive mass, flowering time adaptation to mass loss reduces the final reproductive output of the adapted population, relative to the reproductive output of an unadapted population. On the other hand, when mass loss is restricted to vegetative mass, flowering time adaptation increases the final reproductive output of the adapted population, relative to the reproductive output of an unadapted population. However, because of the asymetric effects of vegetative and reproductive mass loss in a competitive scenario, indiscriminate mass loss also increases the final reproductive output of the adapted population.

When interpreted in the context of climate change (with climate conditions represented by the scenarios explored here), mass loss has its greatest impact on the final reproductive output (overall reduction) of both adapted and unadapted populations.

The asymetry in the effects of vegetative and reproductive mass loss (when they occur simultaneously) can be attributed to the addition of the term representing light competition to the basal production rate, effectively increasing (in late flowering mutants) or decreasing (in early flowering mutants) the relative growth rate, which is directly proportional to vegetative mass. Therefore, under competitive conditions and indiscriminate mass loss, vegetative mass loss has a stronger effect on the flowering time ESS than reproductive mass loss. Without light competition, the effects of vegetative and reproductive mass loss on flowering time ESS cancel out because mass loss in our model is relative and proportional to mass. However, when mass loss is restricted to vegetative mass, its effect is stronger on the flowering time ESS than an equal mass loss rate restricted to reproductive mass because of the longer period of time during which mass loss occurs (vegetative mass loss occurs both before and after flowering, while reproductive mass loss occurs only after flowering).

### 4.1 Model limitations

Our model considers general processes that occur throughout plant growth and which can affect the overall reproductive output of the population and the optimal timing of allocation of resources from vegetative mass growth to reproductive mass growth. Because of this generality, those processes can be interpreted in different ways (e.g., mass loss can be caused by herbivory and/or diseases, and an increase in the basal production rate can be caused by an increase in temperature or CO_2_ level). Despite this generality, there are important limitations that should be considered when comparing our results with future empirical data. For example, we assume that the allocation of resources from vegetative growth to reproductive growth occurs instantaneously, but measured patterns of allocation indicate that the switch to reproduction typically occurs over an interval of a few weeks at least in some species (King & Roughgarden, 1983).

The most important limitation of our model is the constancy of the processes that happen throughout plant life cycle, such as mass production and loss. In real‐world cases, mass production can vary throughout the growth season due to weather conditions as well as variability in the availability of nutrients in the soil. Similarly, mass loss is unlikely to be a quantitatively constant process in nature and will largely depend on the process or agent that causes it. For example, herbivory might only happen during narrow time intervals during plant growth, which limits mass loss to those specific time intervals, generating different questions regarding the fitness effects of the timing of mass loss events relative to flowering time and end of the season.

Another limitation is that the model does not consider the potential dependence on mutualistic interactions, such as plant–pollinator interactions and seed dispersal/germination by herbivores, which might affect the reproductive output of plants and even potentially change the direction of selection for flowering time. Additionally, the formation of seed banks across multiple growth seasons is not explored, but it might be an important process in minimizing the reduction in reproductive output due to detrimental environmental changes (e.g., droughts and fires), or maximizing seed survival under stochastic environmental conditions.

Therefore, the results of our model need to be interpreted under the necessary assumptions that lead to the special cases discussed here. Projections to natural populations should be controled for the processes that are not accounted for in our model, that is, species‐ and population‐specific processes.

### 4.2 Ecological and evolutionary implications

Changes in flowering time can be caused by abiotic or biotic processes and have important implications for community dynamics and agriculture. Under certain conditions, those changes are also followed by changes in plant productivity (final vegetative and/or reproductive mass; Mallikarjuna et al., 2019; Rajendran et al., 2021; Salehi et al., 2005), with direct economic implications for crop plants in particular. Additionally, changes in flowering time can affect community dynamics through the impact of those changes on the species interaction network, which include, e.g., plant–pollinator interactions (Aizen, 2003; Gallagher & Campbell, 2020; Kehrberger & Holzschuh, 2019), seed dispersal via biotic vectors (Aizen, 2003; Segrestin et al., 2018), and other symbiotic interactions.

As previously reported, shifts in the growth season, changes in the growth season duration and changes in the production rate due to, e.g., increased temperature or atmospheric CO_2_ level, are abiotic processes linked to climate change that can lead to changes in flowering time in the long‐term due to selection (Silva et al., 2021). For example, empirical data suggests that an abbreviated growth season caused by drought can lead to the evolution of earlier onset of flowering in *Brassica rapa* (Franks et al., 2007, 2014). Additionally, mass loss, as shown in the current study, can lead to selection for changes in flowering time in either direction, depending on the relative magnitude of vegetative and reproductive mass loss throughout plant growth.

Mass loss can be caused by a wide range of abiotic or biotic processes. Frequent droughts, heat waves and wildfires, for example, are common abiotic effects of climate change that can cause vegetation mass loss (Liu et al., 2021; Wilschut et al., 2022) both at the individual plant level and population level. In contrast, plant diseases caused by viruses, bacteria or fungi, as well as herbivory are common biotic effects that cause vegetation mass loss (Bale et al., 2002; Bebber et al., 2014; Garrett et al., 2006; Pautasso et al., 2012; Ristaino et al., 2021; Singh et al., 2023; Tibpromma et al., 2021). Importantly, climate change can contribute to the spread of infectious diseases in plants and the consequent expansion of pathogen distribution, with the potential to cause cross‐species pathogen spillovers (Laine, 2023; Ristaino et al., 2021; Shaw & Osborne, 2011).

Climate change has emerged as a significant driver of the spread of plant diseases, with profound consequences for plant mass production and food security. It can impact the dynamics of plant diseases through several mechanisms. For example, rising temperatures, altered precipitation patterns and increased atmospheric CO_2_ levels can create more favorable conditions for disease‐causing organisms, enhancing their reproduction and survival. As a consequence, climate change can also expand the geographical ranges of plant pathogens (Laine, 2023; Ristaino et al., 2021; Shaw & Osborne, 2011). Additionally, changes in temperature and humidity can affect the life cycles of plant disease vectors, such as insects (Roos et al., 2011). These altered conditions can result in increased disease prevalence and severity, ultimately leading to mass loss at the individual plant and population levels, affecting agricultural production. Therefore, the cumulative impact of climate change on the dynamics of plant diseases underscores the need for adaptive strategies in agriculture to ensure global food security.

As mentioned above, an additional biotic cause of plant mass loss is herbivory (Bale et al., 2002; Currano et al., 2008; de Sassi & Tylianakis, 2012; Wilf & Labandeira, 1999; Zhang et al., 2019). Climate change can extend the growth season of plants and, as a consequence, the period during which herbivores have access to plant resources, potentially increasing herbivore damage. Moreover, stressors induced by climate change, such as droughts, heatwaves and altered geochemical cycles, can alter the nutritional quality of plants (Christopoulos & Ouzounidou, 2021; Dong et al., 2018), making them more appealing to herbivores, as well as weaken plant immune responses (Coley, 1988; Coley et al., 1985), reducing their ability to defend against herbivores effectively. These combined effects of climate change on plant phenology, plant composition and plant immune defenses can lead to increased herbivory, which may have far‐reaching ecological, agricultural and evolutionary implications.

## 5 CONCLUSION

Flowering time is an important phenological trait in plants and a critical determinant of the success of pollination and fruit or seed development, with immense significance for agriculture as it directly affects crop yield and overall food production. Modeling the emerging abiotic and biotic processes driven by climate change and their effects on the evolution of plant phenology is of paramount importance to understanding how those processes can affect plant communities and ecosystems and mitigating the negative effects of climate change. Additionally, with climate change causing an intensified spread of plant diseases and pests (including herbivory), droughts and wildfires, plant biomass loss has become a growing concern. Therefore, predicting the consequences of different climate‐driven processes to plant phenology in the short‐ and long‐term can help us develop strategies to minimize ecosystemic damage as well as ensure food security.

## AUTHOR CONTRIBUTIONS

**Willian T. A. F. Silva:** Conceptualization (equal); formal analysis (equal); funding acquisition (equal); investigation (equal); methodology (equal); visualization (equal); writing – original draft (equal); writing – review and editing (equal). **Mats Hansson:** Funding acquisition (equal); investigation (equal); writing – original draft (equal); writing – review and editing (equal). **Jacob Johansson:** Conceptualization (equal); formal analysis (equal); funding acquisition (equal); investigation (equal); writing – original draft (equal); writing – review and editing (equal).

## CONFLICT OF INTEREST STATEMENT

The authors declare that they have no conflict of interest.

## Data Availability

All data supporting the findings of this study are available within the paper.

## 1 DERIVATION OF INVASION FITNESS

Let $N_{x}\left(j\right)$ and $N_{y}\left(j\right)$ be the number of adult individuals using the resident and mutant strategies, respectively, and let $N\left(j\right)=N_{x}\left(j\right)+N_{y}\left(j\right)$ be the total population size in year *j*. Let $W\left(x,x,N\left(j\right)\right)$ and $W\left(y,x,N\left(j\right)\right)$ be the *per capita* number of seedlings produced by the resident and mutant strategies, respectively, in a population of size $N\left(j\right)$. It follows that the total number of seedlings produced in year *j* is

(A1)
$$N_{S}\left(j\right)=N_{x}\left(j\right)\cdot W\left(x,x,N\left(j\right)\right)+N_{y}\left(j\right)\cdot W\left(y,x,N\left(j\right)\right)$$

We assume that the proportion of seedlings that survive to the next generation depends on the total number of seedlings $N_{S}\left(j\right)$ produced in the population (regardless of the parental strategy) and the availability of resources (density dependence), such that *K* represents the carrying capacity of the habitat for the species under focus. Therefore, the proportion of seedlings that survive to the next generation is defined as $S\left(N_{S}\left(j\right),K\right)$.

It follows that resident and mutant population growth from year *j* to year *j*+1 can be calculated as follows

(A2)
$$N_{x}\left(j+1\right)=S\left(N_{S}\left(j\right),K\right)\cdot W\left(x,x,N\left(j\right)\right)\cdot N_{x}\left(j\right)$$

(A3)
$$N_{y}\left(j+1\right)=S\left(N_{S}\left(j\right),K\right)\cdot W\left(y,x,N\left(j\right)\right)\cdot N_{y}\left(j\right)$$

Next, we assume that, initially, the mutant strategy is sufficiently rare (adaptive dynamics approach) relative to the resident strategy, that is, $N_{x}\left(j\right)>>N_{y}\left(j\right)$, such that the mutant strategy has a negligible effect on survival. With that, we have

(A4)
$$N_{S}\left(j\right)=N_{x}\left(j\right)\cdot W\left(x,x,N\left(j\right)\right)+N_{y}\left(j\right)\cdot W\left(y,x,N\left(j\right)\right)\approx N_{x}\left(j\right)\cdot W\left(x,x,N\left(j\right)\right)$$

and

(A5)
$$S\left(N_{S}\left(j\right),K\right)\approx S\left(N_{x}\left(j\right)\cdot W\left(x,x,N\left(j\right)\right),K\right)$$

It follows that

(A6)
$$N_{x}\left(j+1\right)=S\left(N_{x}\left(j\right)\cdot W\left(x,x,N\left(j\right)\right),K\right)\cdot W\left(x,x,N\left(j\right)\right)\cdot N_{x}\left(j\right)$$

(A7)
$$N_{y}\left(j+1\right)=S\left(N_{x}\left(j\right)\cdot W\left(x,x,N\left(j\right)\right),K\right)\cdot W\left(y,x,N\left(j\right)\right)\cdot N_{y}\left(j\right)$$

We further assume that seed survival is limited by the availability of resources such that

(A8)
$$S\left(N_{S}\left(j\right),K\right)=\frac{K}{N_{x}\left(j\right)\cdot W\left(x,x,N\left(j\right)\right)}\;\text{if}\;N_{x}\left(j\right)\cdot W\left(x,x,N\left(j\right)\right)>K$$

(A9)
$$S\left(N_{S}\left(j\right),K\right)=1\;\text{when}\;N_{x}\left(j\right)\cdot W\left(x,x,N\left(j\right)\right)\leq K$$

Given (A6) and that $W\left(x,x,N\left(j\right)\right)=h\cdot R_{1}\left(E\right)$, a monomorphic population with a small number of individuals will have positive growth only if $\;W\left(x,x,N\left(j\right)\right)>1$. That implies that extinction will occur if

(A10)
$$R_{1}\left(E\right)<\frac{1}{h}$$

It follows that, as long as the final reproductive mass $R_{1}\left(E\right)$ exceeds the extinction threshold (A10), the population will grow (A9) and, once it reaches the carrying capacity (A8), the total population size will be

(A11)
$$\begin{array}{ll} N_{x}\left(j+1\right) & =S\left(N_{x}\left(j\right)\cdot W\left(x,x,N\left(j\right)\right),K\right)\cdot W\left(x,x,N\left(j\right)\right)\cdot N_{x}\left(j\right)\\ & =\frac{K}{N_{x}\left(j\right)\cdot W\left(x,x,N\left(j\right)\right)}\cdot W\left(x,x,N\left(j\right)\right)\cdot N_{x}\left(j\right)=K \end{array}$$

With that, we conclude that the resident population is at equilibrium when ${N_{x}}^{*}=K$. Given that, the population of a rare mutant strategy in the resident population will grow as follows:

(A12)
$$N_{y}\left(j+1\right)=S\left({N_{x}}^{*}\cdot W\left(x,x,{N_{x}}^{*}\right),K\right)\cdot W\left(y,x,{N_{x}}^{*}\right)\cdot N_{y}\left(j\right)$$

Now let $s_{x}\left(y\right)$ be the invasion fitness of a rare mutant with strategy *y* in a resident population with strategy *x*. Let the invasion fitness be defined as

(A13)
$$s_{x}\left(y\right)=\frac{N_{y}\left(j+1\right)}{N_{y}\left(j\right)}=S\left({N_{x}}^{*}\cdot W\left(x,x,{N_{x}}^{*}\right),K\right)\cdot W\left(y,x,{N_{x}}^{*}\right)$$

We note that, according to (A8) and (A11), the following is true:

(A14)
$$S\left({N_{x}}^{*}\cdot W\left(x,x,{N_{x}}^{*}\right),K\right)=\frac{K}{{N_{x}}^{*}\cdot W\left(x,x,{N_{x}}^{*}\right)}=\frac{1}{W\left(x,x,{N_{x}}^{*}\right)}$$

Therefore, the invasion fitness of the rare mutant can be simplified as

(A15)
$$s_{x}\left(y\right)=\frac{N_{y}\left(j+1\right)}{N_{y}\left(j\right)}=\frac{W\left(y,x\right)}{W\left(x,x\right)}=\frac{R_{2}\left(E\right)}{R_{1}\left(E\right)}$$

## 2 RELATIVE GROWTH RATE AND COMPETITION

We assume that the relative growth rate *P*(*D*(*t*)) is a logistic function of the difference *D*(*t*) between individual vegetative mass $V_{i}\left(t\right)$ and the mean vegetative mass of the population $\bar{V\left(t\right)}$, that is, $D\left(t\right)=V_{i}\left(t\right)-\bar{V\left(t\right)}$. Let *L* be the maximum relative growth rate, *k* be the steepness of the logistic curve and $V_{i}\left(t\right)-\bar{V\left(t\right)}=0$ be the sigmoid's midpoint, representing a monomorphic population where individuals have no competitive advantage or disadvantage relative to the rest of the population. This function can be represented by

(A16)
$$\frac{\mathrm{dP}\left(D\left(t\right)\right)}{\mathrm{dt}}=k\cdot P\left(D\left(t\right)\right)\cdot \left(1-\frac{P\left(D\left(t\right)\right)}{L}\right)$$

which has the solution

(A17)
$$P\left(D\left(t\right)\right)=\frac{L}{1+e^{-k\cdot D\left(t\right)}}$$

We assume that the relative growth rate in a monomorphic population ($D\left(t\right)=0$) is the basal production rate *p*, that is, $P\left(0\right)=\frac{L}{2}=p$. Also, let *c* be the strength of competition, represented by the slope of the curve at the sigmoid's midpoint (monomorphic population), that is, $c={\left.\frac{\mathrm{dP}\left(D\left(t\right)\right)}{\mathrm{dt}}\right|}_{D\left(t\right)=0}$. It follows from (A16) that $c=k\cdot \frac{L}{4}$.

In our model, we use the linearized logistic function above (A17) obtained using Taylor series expansion at $D\left(t\right)=0$, such that

(A18)
$$P\left(D\left(t\right)\right)\approx \frac{P^{\left(0\right)}\left(0\right)}{0!}\cdot {\left(D\left(t\right)-0\right)}^{0}+\frac{P^{\left(1\right)}\left(0\right)}{1!}\cdot {\left(D\left(t\right)-0\right)}^{1}$$

which results in

(A19)
$$P\left(D\left(t\right)\right)\approx p+c\cdot \left(V_{i}\left(t\right)-\bar{V\left(t\right)}\right)$$

Note that in a monomorphic population (resident strategy), we have $P\left(D\left(t\right)\right)\approx p$ because $V_{i}\left(t\right)=\bar{V\left(t\right)}$. Additionally, a rare mutant invading a resident population will grow with the production rate $P\left(D\left(t\right)\right)\approx p+c\cdot \left(V_{i}\left(t\right)-\bar{V\left(t\right)}\right)$.

## 3 GROWTH DYNAMICS AND REPRODUCTIVE OUTPUT OF THE RESIDENT STRATEGY

The resident strategy (subscript “1”) grows as follows:

(A20)
$$\frac{dV_{1}\left(t\right)}{\mathrm{dt}}=\left[p-l_{V}\right]\cdot V_{1}\left(t\right)\;\text{for}\;B\leq t\leq x.$$

(A21)
$$\frac{dV_{1}\left(t\right)}{\mathrm{dt}}=-l_{V}\cdot V_{1}\left(t\right)\;\text{for}\;x<t\leq E.$$

(A22)
$$\frac{dR_{1}\left(t\right)}{\mathrm{dt}}=p\cdot V_{1}\left(t\right)-l_{R}\cdot R_{1}\left(t\right)\;\text{for}\;x<t\leq E.$$

where *x* is the time of allocation of resources (relative growth rate) from vegetative to reproductive growth in the resident strategy, *B* is the beginning of the growth season and *E* is the end of the growth season. Assume $V_{1}\left(B\right)=V_{B}$ and $R_{1}\left(B\right)=0$.

From Equations (A20) and (A21), we obtain the vegetative mass of the resident strategy during the respective time intervals:

(A23)
$$V_{1}\left(t\right)=V_{B}\cdot e^{p\cdot t-l_{V}\cdot t}\;\text{for}\;B<t\leq x.$$

and

(A24)
$$V_{1}\left(t\right)=V_{B}\cdot e^{p\cdot x-l_{V}\cdot t}\;\text{for}\;x<t\leq E.$$

From Equation (A22), we obtain the reproductive mass for *t* > *x* and the final reproductive mass:

(A25)
$$R_{1}\left(t\right)=V_{B}\cdot p\cdot e^{p\cdot x-l_{R}\cdot t}\int_{x}^{t}e^{t\cdot \left(l_{R}-l_{V}\right)}\;\mathrm{dt}\;\text{for}\;x<t\leq E.$$

and

(A26)
$$R_{1}\left(E\right)=V_{B}\cdot p\cdot e^{p\cdot x-l_{R}\cdot E}\int_{x}^{E}e^{t\cdot \left(l_{R}-l_{V}\right)}\;\mathrm{dt}$$

Note that keeping the indefinite integrals in (A25), (A26) and all the subsequent solutions allows us to evaluate the expressions ever when $l_{V}=l_{R}=0$.

The number of seeds produced by the resident strategy that will grow in the next growth season is, thus:

(A27)
$$W\left(x,x\right)=h\cdot V_{B}\cdot p\cdot e^{p\cdot x-l_{R}\cdot E}\int_{x}^{E}e^{t\cdot \left(l_{R}-l_{V}\right)}\;\mathrm{dt}$$

where *h* is a linear factor that converts reproductive mass into number of seeds. Reproductive mass in the resident strategy is maximized when

(A28)
$$e^{x\cdot \left(l_{R}-l_{V}\right)}=p\cdot \int_{x}^{E}e^{t\cdot \left(l_{R}-l_{V}\right)}\;\mathrm{dt}$$

We note that when $l_{V}=l_{R}=0$, (A28) becomes

(A29)
$$x=E-\frac{1}{p}$$

which is in line with the conclusions from previous models by Cohen (1971, 1976), Johansson et al. (2013) and Silva et al. (2021) for the optimal flowering time of the resident strategy (monomorphic population).

If $l_{V}>0$ or $l_{R}>0$, (A28) becomes

(A30)
$$x=\frac{1}{l_{R}-l_{V}}\cdot \ln\left(\frac{p\cdot e^{E\cdot \left(l_{R}-l_{V}\right)}}{l_{R}-l_{V}+p}\right)$$

## 4 GROWTH DYNAMICS AND REPRODUCTIVE OUTPUT OF THE MUTANT STRATEGY

Growth dynamics in the mutant strategy (subscript “2”) occur in different ways depending on its time of allocation of resources (relative growth rate) from vegetative to reproductive growth (*y*) relative to the resident strategy (*x*). As in the resident strategy, assume $V_{2}\left(B\right)=V_{B}$ and $R_{2}\left(B\right)=0$. There are three possible cases:

### 4.1 Case 1: *y* = *x*

When mutant and resident strategies start producing reproductive mass at the same time (*x* = *y*), the mutant strategy grows as follows:

(A31)
$$\frac{dV_{2}}{\mathrm{dt}}=\left[p-l_{V}\right]\cdot V_{2}\left(t\right)\;\text{for}\;B\leq t\leq y.$$

(A32)
$$\frac{dV_{2}}{\mathrm{dt}}=-l_{V}\cdot V_{2}\left(t\right)\;\text{for}\;y<t\leq E.$$

(A33)
$$\frac{dR_{2}}{\mathrm{dt}}=p\cdot V_{2}\left(t\right)-l_{R}\cdot R_{2}\left(t\right)\;\text{for}\;y<t\leq E.$$

Note that in this case, mutant and resident strategies are indistinguishable in their growth dynamics and, thus, we obtain:

(A34)
$$V_{2}\left(t\right)=V_{B}\cdot e^{p\cdot t-l_{V}\cdot t}\;\text{for}\;B<t\leq y.$$

(A35)
$$V_{2}\left(t\right)=V_{B}\cdot e^{p\cdot y-l_{V}\cdot t}\;\text{for}\;y<t\leq E.$$

(A36)
$$R_{2}\left(t\right)=V_{B}\cdot p\cdot e^{p\cdot y-l_{R}\cdot t}\int_{y}^{t}e^{t\cdot \left(l_{R}-l_{V}\right)}\;\mathrm{dt}\;\text{for}\;y<t\leq E.$$

and the final reproductive mass is

(A37)
$$R_{2}\left(E\right)=V_{B}\cdot p\cdot e^{p\cdot y-l_{R}\cdot E}\int_{y}^{E}e^{t\cdot \left(l_{R}-l_{V}\right)}\;\mathrm{dt}$$

It follows that

(A38)
$$W\left(y,x\right)=h\cdot V_{B}\cdot p\cdot e^{p\cdot y-l_{R}\cdot E}\int_{y}^{E}e^{t\cdot \left(l_{R}-l_{V}\right)}\;\mathrm{dt}$$

We note that, because *y* = *x*, we have $W\left(y,x\right)=W\left(x,x\right)$.

### 4.2 Case 2: *y* < *x*

When mutant strategy start producing reproductive mass earlier that the resident strategy (*y* < *x*), the mutant strategy grows as follows:

(A39)
$$\frac{dV_{2}\left(t\right)}{\mathrm{dt}}=\left[p-l_{V}\right]\cdot V_{2}\left(t\right)\;\text{for}\;B\leq t\leq y.$$

(A40)
$$\frac{dV_{2}\left(t\right)}{\mathrm{dt}}=-l_{V}\cdot V_{2}\left(t\right)\;\text{for}\;y<t\leq E.$$

(A41)
$$\frac{dR_{2}\left(t\right)}{\mathrm{dt}}=V_{2}\left(t\right)\cdot \left(p+c\cdot \left[V_{2}\left(t\right)-V_{1}\left(t\right)\right]\right)-l_{R}\cdot R_{2}\left(t\right)\;\text{for}\;y\leq t\leq x.$$

(A42)
$$\frac{dR_{2}\left(t\right)}{\mathrm{dt}}=V_{2}\left(t\right)\cdot \left(p+c\cdot \left[V_{2}\left(t\right)-V_{1}\left(t\right)\right]\right)-l_{R}\cdot R_{2}\left(t\right)\;\text{for}\;x<t\leq E.$$

From Equations (A39) and (A40), we obtain

(A43)
$$V_{2}\left(t\right)=V_{B}\cdot e^{p\cdot t-l_{V}\cdot t}\;\text{for}\;B\leq t\leq y.$$

and

(A44)
$$V_{2}\left(t\right)=V_{B}\cdot e^{p\cdot y-l_{V}\cdot t}\;\text{for}\;y<t\leq E.$$

From Equations (A41) and (A42), we obtain

(A45)
$$\begin{array}{c} R_{2}\left(t\right)=V_{B}\cdot e^{p\cdot y-l_{R}\cdot t}\cdot \\ \left[p\cdot \int_{y}^{t}e^{t\cdot \left(l_{R}-l_{V}\right)}\;\mathrm{dt}+c\cdot V_{B}\cdot \left(e^{p\cdot y}\cdot \int_{y}^{t}e^{t\cdot \left(l_{R}-2\cdot l_{V}\right)}\;\mathrm{dt}-\int_{y}^{t}e^{t\cdot \left(p+l_{R}-2\cdot l_{V}\right)}\;\mathrm{dt}\right)\right]\\ \text{for}\;y\leq t\leq x. \end{array}$$

and

(A46)
$$R_{2}\left(t\right)=V_{B}\cdot e^{p\cdot y-l_{R}\cdot t}\cdot \left[p\cdot \int_{y}^{t}e^{t\cdot \left(l_{R}-l_{V}\right)}\;\mathrm{dt}+c\cdot V_{B}\cdot \left(e^{p\cdot y}\cdot \int_{y}^{x}e^{t\cdot \left(l_{R}-2\cdot l_{V}\right)}\;\mathrm{dt}-\int_{y}^{x}e^{t\cdot \left(p+l_{R}-2\cdot l_{V}\right)}\;\mathrm{dt}+\left(e^{p\cdot y}-e^{p\cdot x}\right)\cdot \int_{x}^{t}e^{t\cdot \left(l_{R}-2\cdot l_{V}\right)}\;\mathrm{dt}\right)\right]\;\text{for}\;x\leq t\leq E.$$

and the final reproductive mass is

(A47)
$$R_{2}\left(E\right)=V_{B}\cdot e^{p\cdot y-l_{R}\cdot E}\cdot \left[p\cdot \int_{y}^{E}e^{t\cdot \left(l_{R}-l_{V}\right)}\;\mathrm{dt}+c\cdot V_{B}\cdot \left(e^{p\cdot y}\cdot \int_{y}^{x}e^{t\cdot \left(l_{R}-2\cdot l_{V}\right)}\;\mathrm{dt}-\int_{y}^{x}e^{t\cdot \left(p+l_{R}-2\cdot l_{V}\right)}\;\mathrm{dt}+\left(e^{p\cdot y}-e^{p\cdot x}\right)\cdot \int_{x}^{E}e^{t\cdot \left(l_{R}-2\cdot l_{V}\right)}\;\mathrm{dt}\right)\right]$$

It follows that

(A48)
$$W\left(y,x\right)=h\cdot V_{B}\cdot e^{p\cdot y-l_{R}\cdot E}\cdot \left[p\cdot \int_{y}^{E}e^{t\cdot \left(l_{R}-l_{V}\right)}\;\mathrm{dt}+c\cdot V_{B}\cdot \left(e^{p\cdot y}\cdot \int_{y}^{x}e^{t\cdot \left(l_{R}-2\cdot l_{V}\right)}\;\mathrm{dt}-\int_{y}^{x}e^{t\cdot \left(p+l_{R}-2\cdot l_{V}\right)}\;\mathrm{dt}+\left(e^{p\cdot y}-e^{p\cdot x}\right)\cdot \int_{x}^{E}e^{t\cdot \left(l_{R}-2\cdot l_{V}\right)}\;\mathrm{dt}\right)\right]$$

### 4.3 Case 3: *y* > *x*

When mutant strategy start producing reproductive mass later than the resident strategy (*y* > *x*), the mutant strategy grows as follows:

(A49)
$$\frac{dV_{2}\left(t\right)}{\mathrm{dt}}=\left[p-l_{V}\right]\cdot V_{2}\left(t\right)\;\text{for}\;B\leq t\leq x.$$

(A50)
$$\frac{dV_{2}\left(t\right)}{\mathrm{dt}}=V_{2}\left(t\right)\cdot \left(p-l_{V}+c\cdot \left[V_{2}\left(t\right)-V_{1}\left(t\right)\right]\right)\;\text{for}\;x<t\leq y.$$

(A51)
$$\frac{dV_{2}\left(t\right)}{\mathrm{dt}}=-l_{V}\cdot V_{2}\left(t\right)\;\text{for}\;y<t\leq E.$$

(A52)
$$\frac{dR_{2}\left(t\right)}{\mathrm{dt}}=V_{2}\left(t\right)\cdot \left(p+c\cdot \left[V_{2}\left(t\right)-V_{1}\left(t\right)\right]\right)-l_{R}\cdot R_{2}\left(t\right)\;\text{for}\;y<t\leq E.$$

From Equations (A49), (A50) and (A51), we obtain

(A53)
$$V_{2}\left(t\right)=V_{B}\cdot e^{p\cdot t-l_{V}\cdot t}\;\text{for}\;B<t\leq x.$$

and

(A54)
$$V_{2}\left(t\right)=\frac{V_{B}\cdot e^{p\cdot t-l_{V}\cdot t+c\cdot V_{B}\cdot e^{p\cdot x}\cdot \left({\left.\int e^{-l_{V}\cdot t}\;\mathrm{dt}\right|}_{t=x}-\int e^{-l_{V}\cdot t}\;\mathrm{dt}\right)}}{1-c\cdot V_{B}\cdot e^{c\cdot V_{B}\cdot e^{p\cdot x}\cdot {\left.\int e^{-l_{V}\cdot t}\;\mathrm{dt}\right|}_{t=x}}\cdot \int_{x}^{t}e^{p\cdot t-l_{V}\cdot t-c\cdot V_{B}\cdot e^{p\cdot x}\cdot \int e^{-l_{V}\cdot t}\;\mathrm{dt}}\;\mathrm{dt}}\;\text{for}\;x<t\leq y.$$

and

(A55)
$$V_{2}\left(t\right)=\frac{V_{B}\cdot e^{p\cdot y-l_{V}\cdot t+c\cdot V_{B}\cdot e^{p\cdot x}\cdot \left({\left.\int e^{-l_{V}\cdot t}\;\mathrm{dt}\right|}_{t=x}-{\left.\int e^{-l_{V}\cdot t}\;\mathrm{dt}\right|}_{t=y}\right)}}{1-c\cdot V_{B}\cdot e^{c\cdot V_{B}\cdot e^{p\cdot x}\cdot {\left.\int e^{-l_{V}\cdot t}\;\mathrm{dt}\right|}_{t=x}}\cdot \int_{x}^{y}e^{p\cdot t-l_{V}\cdot t-c\cdot V_{B}\cdot e^{p\cdot x}\cdot \int e^{-l_{V}\cdot t}\;\mathrm{dt}}\;\mathrm{dt}}\;\text{for}\;y<t\leq E.$$

From Equation (A52), we obtain

(A56)
$$R_{2}\left(t\right)=\frac{V_{B}\cdot e^{p\cdot y-l_{R}\cdot t+c\cdot V_{B}\cdot e^{p\cdot x}\cdot \left({\left.\int e^{-l_{V}\cdot t}\;\mathrm{dt}\right|}_{t=x}-{\left.\int e^{-l_{V}\cdot t}\;\mathrm{dt}\right|}_{t=y}\right)}}{1-c\cdot V_{B}\cdot e^{c\cdot V_{B}\cdot e^{p\cdot x}\cdot {\left.\int e^{-l_{V}\cdot t}\;\mathrm{dt}\right|}_{t=x}}\cdot \int_{x}^{y}e^{p\cdot t-l_{V}\cdot t-c\cdot V_{B}\cdot e^{p\cdot x}\cdot \int e^{-l_{V}\cdot t}\;\mathrm{dt}}\;\mathrm{dt}}\cdot \left[p\cdot \int_{y}^{t}e^{t\cdot \left(l_{R}-l_{V}\right)}\;\mathrm{dt}+c\cdot \int_{y}^{t}e^{t\cdot \left(l_{R}-2\cdot l_{V}\right)}\;\mathrm{dt}\cdot \left[\frac{V_{B}\cdot e^{p\cdot y+c\cdot V_{B}\cdot e^{p\cdot x}\cdot \left({\left.\int e^{-l_{V}\cdot t}\;\mathrm{dt}\right|}_{t=x}-{\left.\int e^{-l_{V}\cdot t}\;\mathrm{dt}\right|}_{t=y}\right)}}{1-c\cdot V_{B}\cdot e^{c\cdot V_{B}\cdot e^{p\cdot x}\cdot {\left.\int e^{-l_{V}\cdot t}\;\mathrm{dt}\right|}_{t=x}}\cdot \int_{x}^{y}e^{p\cdot t-l_{V}\cdot t-c\cdot V_{B}\cdot e^{p\cdot x}\cdot \int e^{-l_{V}\cdot t}\;\mathrm{dt}}\;\mathrm{dt}}-V_{B}\cdot e^{p\cdot x}\right]\right]\;\text{for}\;y<t\leq E.$$

and the final reproductive mass is

(A57)
$$\begin{array}{c} R_{2}\left(E\right)=\frac{V_{B}\cdot e^{p\cdot y-l_{R}\cdot E+c\cdot V_{B}\cdot e^{p\cdot x}\cdot \left({\left.\int e^{-l_{V}\cdot t}\;\mathrm{dt}\right|}_{t=x}-{\left.\int e^{-l_{V}\cdot t}\;\mathrm{dt}\right|}_{t=y}\right)}}{1-c\cdot V_{B}\cdot e^{c\cdot V_{B}\cdot e^{p\cdot x}\cdot {\left.\int e^{-l_{V}\cdot t}\;\mathrm{dt}\right|}_{t=x}}\cdot \int_{x}^{y}e^{p\cdot t-l_{V}\cdot t-c\cdot V_{B}\cdot e^{p\cdot x}\cdot \int e^{-l_{V}\cdot t}\;\mathrm{dt}}\;\mathrm{dt}}\cdot \\ \left[p\cdot \int_{y}^{E}e^{t\cdot \left(l_{R}-l_{V}\right)}\;\mathrm{dt}+c\cdot \int_{y}^{E}e^{t\cdot \left(l_{R}-2\cdot l_{V}\right)}\;\mathrm{dt}\cdot \left[\frac{V_{B}\cdot e^{p\cdot y+c\cdot V_{B}\cdot e^{p\cdot x}\cdot \left({\left.\int e^{-l_{V}\cdot t}\;\mathrm{dt}\right|}_{t=x}-{\left.\int e^{-l_{V}\cdot t}\;\mathrm{dt}\right|}_{t=y}\right)}}{1-c\cdot V_{B}\cdot e^{c\cdot V_{B}\cdot e^{p\cdot x}\cdot {\left.\int e^{-l_{V}\cdot t}\;\mathrm{dt}\right|}_{t=x}}\cdot \int_{x}^{y}e^{p\cdot t-l_{V}\cdot t-c\cdot V_{B}\cdot e^{p\cdot x}\cdot \int e^{-l_{V}\cdot t}\;\mathrm{dt}}\;\mathrm{dt}}-V_{B}\cdot e^{p\cdot x}\right]\right] \end{array}$$

It follows that

(A58)
$$\begin{array}{c} W\left(y,x\right)=h\cdot \frac{V_{B}\cdot e^{p\cdot y-l_{R}\cdot E+c\cdot V_{B}\cdot e^{p\cdot x}\cdot \left({\left.\int e^{-l_{V}\cdot t}\;\mathrm{dt}\right|}_{t=x}-{\left.\int e^{-l_{V}\cdot t}\;\mathrm{dt}\right|}_{t=y}\right)}}{1-c\cdot V_{B}\cdot e^{c\cdot V_{B}\cdot e^{p\cdot x}\cdot {\left.\int e^{-l_{V}\cdot t}\;\mathrm{dt}\right|}_{t=x}}\cdot \int_{x}^{y}e^{p\cdot t-l_{V}\cdot t-c\cdot V_{B}\cdot e^{p\cdot x}\cdot \int e^{-l_{V}\cdot t}\;\mathrm{dt}}\;\mathrm{dt}}\cdot \\ \left[p\cdot \int_{y}^{E}e^{t\cdot \left(l_{R}-l_{V}\right)}\;\mathrm{dt}+c\cdot \int_{y}^{E}e^{t\cdot \left(l_{R}-2\cdot l_{V}\right)}\;\mathrm{dt}\cdot \left[\frac{V_{B}\cdot e^{p\cdot y+c\cdot V_{B}\cdot e^{p\cdot x}\cdot \left({\left.\int e^{-l_{V}\cdot t}\;\mathrm{dt}\right|}_{t=x}-{\left.\int e^{-l_{V}\cdot t}\;\mathrm{dt}\right|}_{t=y}\right)}}{1-c\cdot V_{B}\cdot e^{c\cdot V_{B}\cdot e^{p\cdot x}\cdot {\left.\int e^{-l_{V}\cdot t}\;\mathrm{dt}\right|}_{t=x}}\cdot \int_{x}^{y}e^{p\cdot t-l_{V}\cdot t-c\cdot V_{B}\cdot e^{p\cdot x}\cdot \int e^{-l_{V}\cdot t}\;\mathrm{dt}}\;\mathrm{dt}}-V_{B}\cdot e^{p\cdot x}\right]\right] \end{array}$$

## 5 SINGULAR POINTS

As noted above and by Silva et al. (2021), the invasion fitness of the mutant strategy relative to the resident strategy is $s_{x}\left(y\right)=\frac{W\left(y,x\right)}{W\left(x,x\right)}$. The singular point (ESS) is the strategy that, as a resident strategy, cannot be invaded by any mutant. It is found when

(A59)
$${\left.\frac{ds_{x}\left(y\right)}{\mathrm{dy}}\right|}_{y=x}=0$$

We note that $W\left(x,x\right)$ (A27) can be treated as a non‐zero constant that does not depend on the mutant strategy. Because of that, $\frac{ds_{x}\left(y\right)}{\mathrm{dy}}=\frac{1}{W\left(x,x\right)}\cdot \frac{\mathrm{dW}\left(y,x\right)}{\mathrm{dy}}$ and, thus, the singular point is found when ${\left.\frac{\mathrm{dW}\left(y,x\right)}{\mathrm{dy}}\right|}_{y=x}=0$.

For case 1 (*y* = *x*), we note that *W*(*y*,*x*) = *W*(*x*,*x*) and the invasion fitness is $s_{x}\left(y\right)=\frac{W\left(y,x\right)}{W\left(x,x\right)}=\frac{W\left(x,x\right)}{W\left(x,x\right)}=1$. It follows that ${\left.\frac{ds_{x}\left(x\right)}{\mathrm{dx}}\right|}_{y=x}=0$ for all *y* = *x*. For cases 2 (*y* < *x*) and 3 (*y* > *x*), the singular point is found by solving Equation (A59). Because the singular point does not depend on any particular mutant strategy, it can be solved for in either case. Therefore, for *y* ≠ *x*, the singular point is the value of *x** that solves the equation

(A60)
$$e^{x^{*}\cdot \left(l_{R}-l_{V}\right)}=c\cdot V_{B}\cdot e^{p\cdot x^{*}}\cdot \int_{x^{*}}^{E}e^{t\cdot \left(l_{R}-2\cdot l_{V}\right)}\;\mathrm{dt}+p\cdot \int_{x^{*}}^{E}e^{t\cdot \left(l_{R}-l_{V}\right)}\;\mathrm{dt}$$

As long as $l_{R}-l_{V}\neq 0$, the singular point (A60) can be defined as the value of *x** that solves the equation

(A61)
$$x^{*}=\frac{1}{l_{R}-l_{V}}\cdot \ln\left(c\cdot V_{B}\cdot e^{p\cdot x^{*}}\cdot \int_{x^{*}}^{E}e^{t\cdot \left(l_{R}-2\cdot l_{V}\right)}\;\mathrm{dt}+p\cdot \int_{x^{*}}^{E}e^{t\cdot \left(l_{R}-l_{V}\right)}\;\mathrm{dt}\right)$$

We note that in the absence of assymetric competition (*c* = 0), the singular point (ESS) is the strategy that maximizes the final reproductive mass in the resident population, defined as

(A62)
$$x^{*}=E-\frac{1}{l_{R}-l_{V}}\cdot \ln\left(1+\frac{l_{R}-l_{V}}{p}\right)$$

and that

(A63)
$$\frac{dx^{*}}{dl_{V}}=\frac{1}{{\left(l_{R}-l_{V}\right)}^{2}}\cdot \left[\frac{l_{R}-l_{V}}{l_{R}-l_{V}+p}-\ln\left(\frac{l_{R}-l_{V}+p}{p}\right)\right]$$

(A64)
$$\frac{dx^{*}}{dl_{R}}=\frac{1}{{\left(l_{R}-l_{V}\right)}^{2}}\cdot \left[\ln\left(\frac{l_{R}-l_{V}+p}{p}\right)-\frac{l_{R}-l_{V}}{l_{R}-l_{V}+p}\right]$$

It follows that $\frac{dx^{*}}{dl_{V}}>0$ if $\ln\left(\frac{l_{R}-l_{V}+p}{p}\right)<\frac{l_{R}-l_{V}}{l_{R}-l_{V}+p}$ and $\frac{dx^{*}}{dl_{V}}<0$ if $\ln\left(\frac{l_{R}-l_{V}+p}{p}\right)>\frac{l_{R}-l_{V}}{l_{R}-l_{V}+p}$. Similarly, $\frac{dx^{*}}{dl_{R}}>0$ if $\ln\left(\frac{l_{R}-l_{V}+p}{p}\right)>\frac{l_{R}-l_{V}}{l_{R}-l_{V}+p}$ and $\frac{dx^{*}}{dl_{R}}<0$ if $\ln\left(\frac{l_{R}-l_{V}+p}{p}\right)<\frac{l_{R}-l_{V}}{l_{R}-l_{V}+p}$. In other words, the ESS will respond in different directions depending on the relationship between vegetative and reproductive mass loss and the basal mass production rate.

In the absence of mass loss ($l_{V}=l_{R}=0$), the singular point is the same found by Silva et al. (2021)

(A65)
$$x^{*}=E-\frac{1}{p+c\cdot V_{B}\cdot e^{p\cdot x^{*}}}$$

which becomes $x^{*}=E-\frac{1}{p}$ in the absence of competition. The singular points maximize invasion fitness and, therefore, represent evolutionary stable strategies (ESS) that, as resident strategies, cannot be invaded.
